# Supplementary material for: Dronedarone hydrochloride targets cardiolipin and phosphatidylglycerol to increase colistin susceptibility in gram-negative pathogens
Source: Microbiol Spectr. 2025 Sep 30;13(11):e01196-25. doi: 10.1128/spectrum.01196-25 (PMC12584652; doi:10.1128/spectrum.01196-25)
Supplement: Supplemental material — Figures S1 to S3; Table S1. [file spectrum.01196-25-s0001.docx]

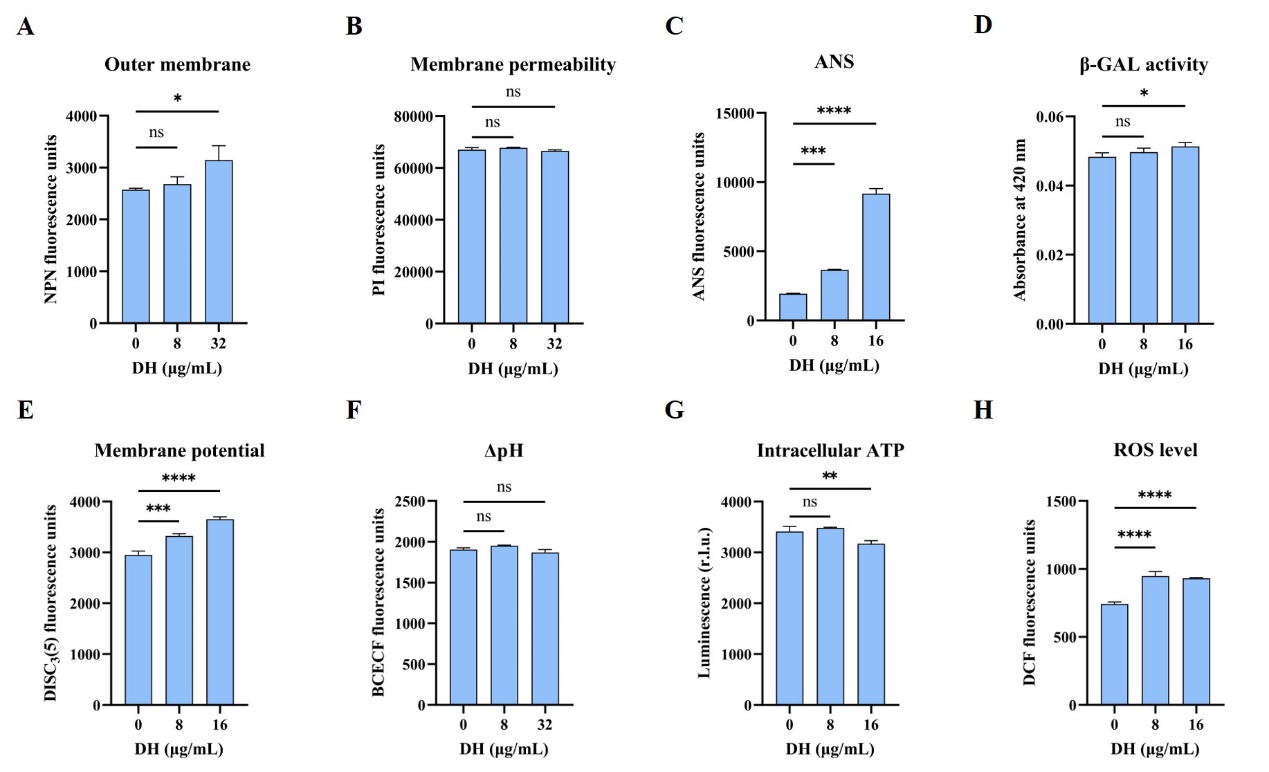


**Figure S1. DH disrupts bacterial cellular metabolism.** Outer membrane **(A)** and inner membrane **(B)** permeability changes of *E. coli* ECQ001 treated with DH. **(C)** Membrane fluidity change of *E. coli* ECQ001 treated with DH. **(D)** β-GAL release of *E. coli* ECQ001 treated with DH. Membrane potential **(E)** and ΔpH change **(F)** of the inner membrane in *E. coli* ECQ001. Intracellular ATP **(G)** and ROS **(H)** levels of *E. coli* ECQ001 treated with DH. Data are expressed as mean ± SD. ns, no significance, ^∗^*P* < 0.05, ^∗∗^*P* < 0.01, ^∗∗∗^*P* < 0.001, ^∗∗∗∗^*P* < 0.0001 by one-way ANOVA.


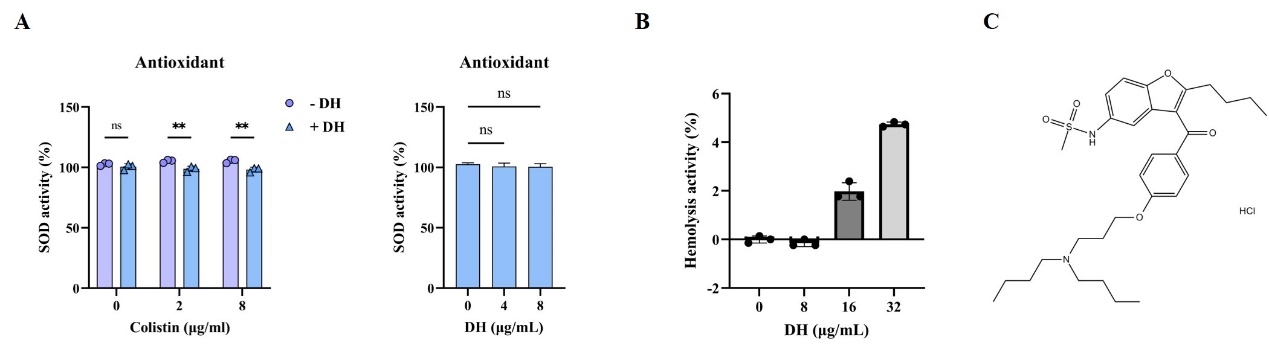


**Figure S2. Antioxidant Levels and Hemolytic Activity. (A)** SOD activity of *E. coli* ECQ001 after exposure to DH, colistin, or the combination. **(B)** Hemolytic activity of DH on the red blood cells of sheep. (C) Structure of DH. Data are expressed as mean ± SD. Ns indicates no significance, and ^∗∗^*P* < 0.01 by one-way ANOVA.


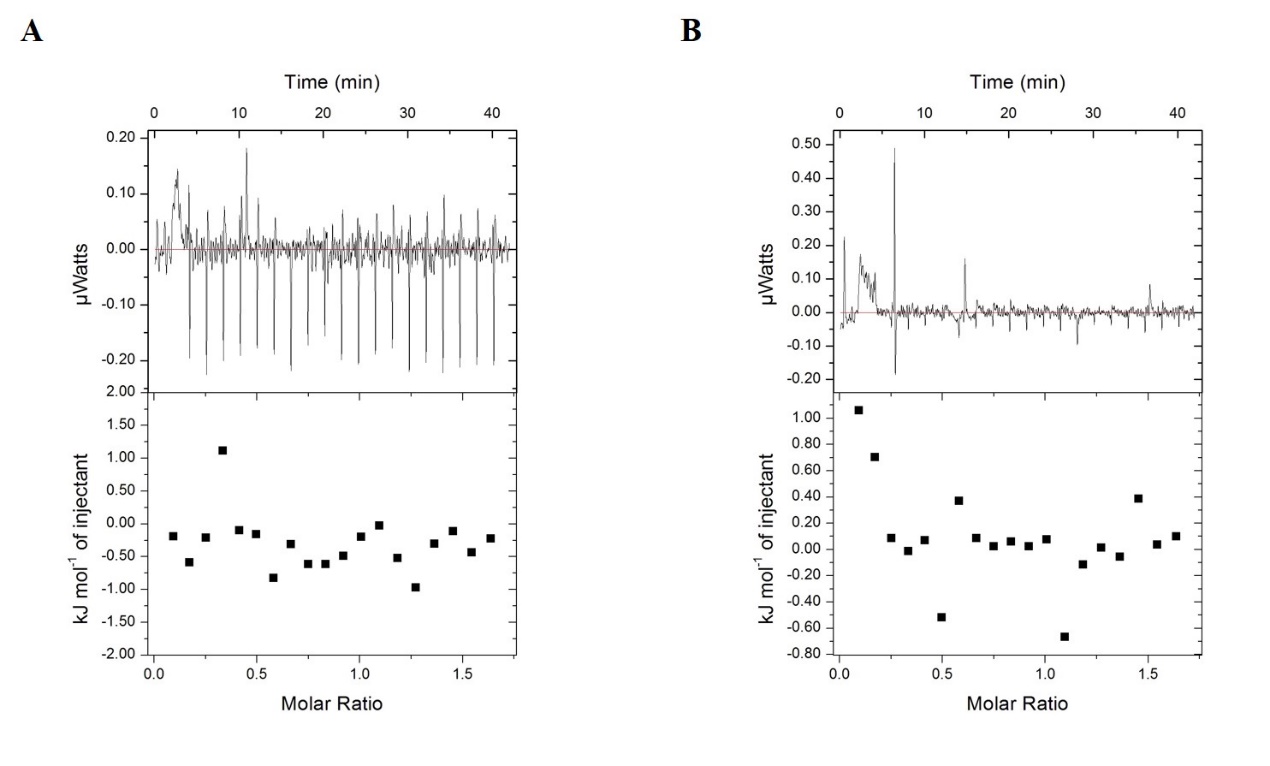


**Figure S3. Binding affinity of DH. (A)** The affinity between DH and PE. **(B)** The affinity between DH and buffer.

**Supplementary Table 1 Bacterial strains used in this study.**

| Strains | Colistin Resistance | *mcr* confirmation | Source/Reference |
| --- | --- | --- | --- |
| **Gram-negative bacteria** |  |  |  |
| *E. coli* ATCC 25922 | - | - | ATCC 25922 |
| ExPEC ECQ001 | **+** | - | In this study |
| ExPEC 42 | **+** | - | [1] |
| ExPEC 1145 | **+** | - | [1] |
| ExPEC 1209 | **+** | - | [1] |
| *E. coli* B2 | **+** | **+** | [2] |
| *E. coli* DH5α-PME6032 | - | - | Laboratory strain |
| *E. coli* DH5α-PME6032+*mcr-1* | **+** | **+** | Laboratory strain |
| *K. pneumoniae* ATCC 70063 | - | - | ATCC 70063 |
| *K. pneumoniae* ZJ02 | **+** | **+** | [3] |
| *S.* Typhimurium SL1344 | - | - | [4] |
| *S.* Typhimurium HYM2 | **+** | **+** | Laboratory strain |
| *A. baumannii* ATCC 17978 | - | - | ATCC 17978 |
| **Gram-positive bacteria** |  |  |  |
| *S. aureus* Newman |  | - | Laboratory strain |

ATCC, American Type Culture Collection.

1. Tan, C., et al., *Genome Sequence of a Porcine Extraintestinal Pathogenic Escherichia coli Strain.* Journal of Bacteriology, 2011. **193**(18): p. 5038-5038.

2. Song, M., et al., *A broad-spectrum antibiotic adjuvant reverses multidrug-resistant Gram-negative pathogens.* Nat Microbiol, 2020. **5**(8): p. 1040-1050.

3. Zhou, Y., et al., *Discovery of a potential MCR-1 inhibitor that reverses polymyxin activity against clinical mcr-1-positive Enterobacteriaceae.* Journal of Infection, 2019. **78**(5): p. 364-372.

4. Li, Z., et al., *<i>Salmonella</i> Proteomic Profiling during Infection Distinguishes the Intracellular Environment of Host Cells.* mSystems, 2019. **4**(2): p. 10.1128/msystems.00314-18.

**References**
